# Thoracic endovascular aortic repair of an anastomosis pseudoaneurysm after the Bentall procedure assisted by rapid ventricular pacing: A case report

**DOI:** 10.1016/j.heliyon.2023.e16833

**Published:** 2023-05-31

**Authors:** Jia-Piao Lin, Hui Zhang, Tao Shang, Bing-Xin Jin, Yong-Xing Yao

**Affiliations:** a Department of Anesthesia, First Affiliated Hospital, Zhejiang University School of Medicine, Hangzhou, People's Republic of China; b Department of Vascular Surgery, First Affiliated Hospital, Zhejiang University School of Medicine, Hangzhou, People's Republic of China

**Keywords:** Thoracic endovascular aortic repair, TEVAR, Pseudoaneurysm, Rapid ventricular pacing, RVP

## Abstract

**Background:**

Although commonly used for the treatment of descending aortic dissection, endovascular repair is challenging for ascending aortic pseudoaneurysms. Rapid ventricular pacing (RVP), a method that temporarily impedes cardiac output by stopping ventricular activity, heralds potential benefits for thoracic endovascular aortic repair (TEVAR) during precision landing. Recently, we successfully treated an anastomosis pseudoaneurysm after the Bentall procedure using TEVAR assisted by RVP.

**Case report:**

A 69-year-old male was admitted to our hospital with a ascending aortic anastomosis pseudoaneurysm. He had undergone a Bentall procedure and a coronary artery bypass grafting nine years prior. After extensive consultation, the decision was made to perform TEVAR with the assistance of RVP. After a covered stent graft was delivered to the precise location of the ascending aorta, RVP was performed at a frequency of 180 beats/min with a pacemaker. When a flattened arterial blood wave of <50 mmHg was observed, the stent graft was released precisely between the opening of the coronary graft and innominate artery. Angiography revealed the presence of an endoleak; therefore, a set of interlock coils were packed into the aneurysm. Subsequent angiography showed intact blood flow in the aorta, superior arch branches, and coronary graft vessels. The patient recovered uneventfully after the procedure. He was discharged six days later and was doing well at the eight-month follow-up.

**Conclusion:**

The case indicates that TEVAR assisted by RVP is a promising combination for ascending aortic pseudoaneurysm in selected patients.

## 1 Introduction

Ascending aortic pseudoaneurysms are generally asymptomatic until they present with severe complications of dissection or rupture; therefore, surgical intervention is usually required when the diagnosis is established [1]. Although open chest revascularization is a widely performed procedure for the treatment of ascending aortic pseudoaneurysms, it still carries a high risk of morbidity and mortality [2,3]. Thoracic endovascular aortic repair (TEVAR) —a mature technique for the treatment of descending aortic pathologies —has emerged as an alternative option, and seems to have potential advantages for ascending aortic lesions [4]. However, due to anatomic and physiologic issues, TEVAR for ascending lesions is much more challenging than for descending pathologies. Any imprecise landing of the stent graft can lead to lethal complications, such as coronary artery or brachiocephalic vessel obstruction [5]. Transiently diminishing the blood flow during landing and anchoring might decrease the incidence of these complication [4]. Rapid ventricular pacing (RVP), originating from balloon valvuloplasty and valve deployment, is a perfect method that can temporarily impedes cardiac output by stopping ventricular activity [6]. Recently, it has been frequently used in transcatheter aortic valve replacement [7]. Reports revealed that RVP may have potential benefits in TEVAR during precision landing and stent graft release for ascending aortic lesions [8]. Here, we describe a ascending aortic anastomosis pseudoaneurysm after the Bentall procedure that was successfully treated with TEVAR assisted by RVP.

## 2 Case report

This case was reviewed by the ethical committee of our hospital, and informed consent was obtained from the patient that all images and clinical data and other data included in the manuscript to be published. A 69-year-old man was admitted to our hospital with a ascending aortic pseudoaneurysm at the distal anastomosis of the Bentall graft identified via computed tomography angiography (CTA). He was asymptomatic. Nine years prior, the patient experienced chest pain and was diagnosed with a DeBakey type I aortic dissection involving the coronary artery. Therefore, he received a Bentall procedure, which includes an aortic root replacement, a mechanical aortic valve replacement, and a right coronary artery graft with the great saphenous vein.

**Fig. 1:**
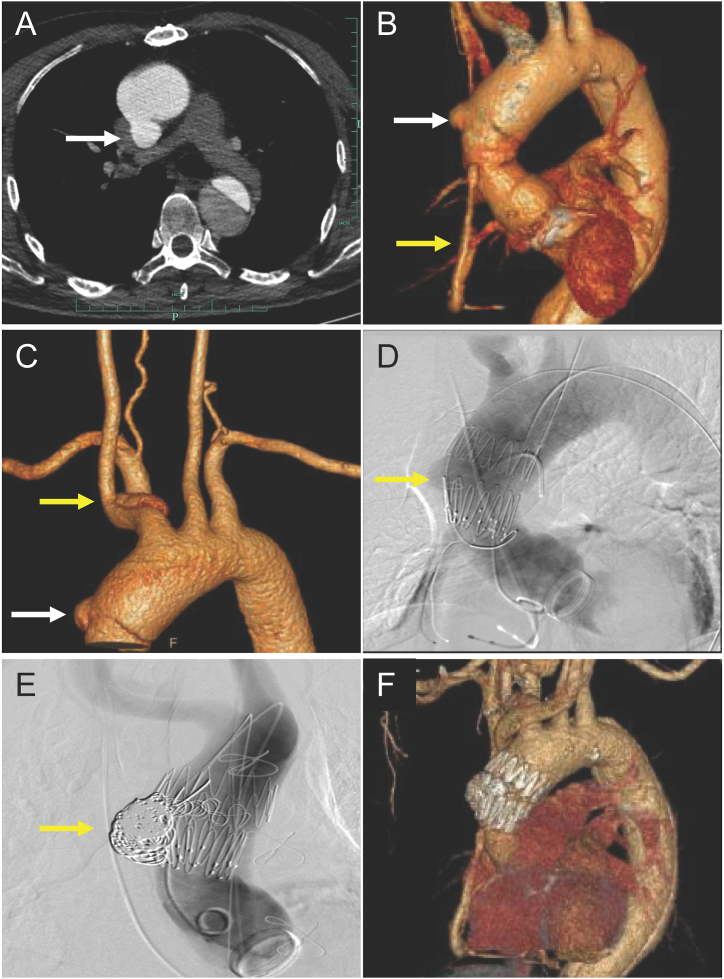
Images showing the anastomosis pseudoaneurysm and the stent graft. **A**. Computed tomography angiography showing the transverse view of the pseudoaneurysm (white arrow). **B**. Three-dimensional reconstruction showing the pseudoaneurysm (white arrow) and the coronary artery bypass graft (yellow arrow). **C**. Three-dimensional reconstruction showing the pseudoaneurysm (white arrow) and a residual false lumen in the brachiocephalic artery (yellow arrow). **D**. Angiography image showing the stent graft location and the endoleak (yellow arrow). **E**. Angiography showing the interlock coils (yellow arrow) packed into the pseudoaneurysm. **F**. Follow-up computed tomography reconstruction showing the stent graft in situ. (For interpretation of the references to colour in this figure legend, the reader is referred to the Web version of this article.)

On admission, a physical examination revealed no abnormalities. His electrocardiogram demonstrated sinus rhythm and a first-degree atrioventricular block. Echocardiography showed mild tricuspid valve regurgitation, decreased left ventricular diastolic function (with a global ejection fraction of 69%), normal blood flow in artificial blood vessels, and normal aortic valve function. CTA revealed a pseudoaneurysm at the distal anastomosis of the graft in the ascending aorta. The diameter of the pseudoaneurysm was around 20 mm, with a residual false lumen in the brachiocephalic artery; the coronary artery graft vessel was smooth without obvious stenosis (Fig. 1A, B, C). Laboratory results showed his hemoglobin was 147 g/L, albumin 48.2 g/L, and alanine transaminase 10 u/L. His serum troponin I was 0.022 ng/mL.

After extensive consultation, the surgical team decided to perform TEVAR with the assistance of RVP. Under standard monitoring, general anesthesia was performed. Mechanical ventilation was adjusted to maintain an end-tidal carbon dioxide partial pressure of 35–40 mmHg. Norepinephrine (2–5 μg/min) was infused to maintain the hemodynamics. After internal jugular vein catheterization, a bipolar balloon-floating pacing catheter (BP2502-10; Biosensors International Pte. Ltd., Singapore) was advanced into the right ventricle via an 8.5F introducer (St. Jude Medical Inc., Saint Paul, Minnesota, USA). X-ray was used to confirm the correct positioning. The function was meticulously tested using a pacemaker (5348; Medtronic Inc., Minnesota, USA).

Next, the bilateral femoral, left carotid, and left brachial arteries were exposed. A 4F sheath was inserted into the left carotid, and a 4F multipurpose angiographic (MPA) catheter was passed into the pseudoaneurysm. A long 8F sheath was used to gain access to the coronary bypass vessel from the left brachial artery in the same way for a potential stent in the bypass. The right femoral artery approach was to deploy the stent graft over the stiff guidewire, while a 4F pigtail catheter for angiography was inserted on the contralateral side. A guidewire (TSMG-35-260-LES, 0.035/260 cm; Cook Medical, Bloomington, Indiana, USA) was inserted through the right external iliac artery, and two other guide wires were placed into the coronary graft and aneurysm through the left brachial artery and carotid artery, respectively. Angiography confirmed the pseudoaneurysm located at the distal anastomosis of the artificial vessel of the ascending aorta. The celiac trunk and superior mesenteric arteries were supplied by the true lumen, while the left and right renal arteries were supplied by both the true and false lumens. Repeated angiographies were performed to confirm that the guide wire passed through the true lumen.

The adopted endograft was modified by the surgeons based on the Zenith Thoracic Endovascular graft (42 mm diameter; Cook Medical, USA). The physician cut it to 4 cm in length without proximal bare spring. The tip of the delivery system was also shortened so that the stent-graft could reach the originating point of the coronary bypass. When the graft was delivered to the ascending aorta, RVP was started at a frequency of 180 beats/min in the ventricular inhibited mode (sensitivity: 5 mV, output: 7 mA). As soon as the monitor showed a flattened arterial blood wave <50 mmHg, the stent graft was released precisely between the coronary bridge vessel and the innominate artery. The proximal end of the stent graft was in close proximity to the coronary bridge vessel, and the distal end was 10 mm anterior to the innominate artery. After stopping the RVP, the hemodynamics stabilized. The duration of RVP was about 30 seconds. Subsequent angiography revealed the presence of an endoleak; therefore, 13 coils (M001363830, 20 mm/40 cm, Interlock; Boston Scientific Corporation, Marlborough, Massachusetts, USA) were packed into the aneurysm through the preset guidewire.

Finally, angiography revealed that the leakage was blocked, with an intact blood flow in the aorta, superior arch branches, and coronary graft vessels (Fig. 1D and E). The procedure took 221 minutes, and the estimated blood loss was 200 mL. The patient was extubated for 17 h and returned to the general ward 2 days after the operation. Six days later, the patient was discharged without any complications. He was doing well postoperatively. Contrast CT showed the stent graft was in situ 8 months later (Fig. 1F).

## 3 Discussion

The anatomical and physiological features of the aorta lead to the ascending and arch aortic lesions are particular difficult to treat with endovascular technology. In medical practices, most pathologies of the ascending aorta—including aneurysms, dissections, intramural hematomas, and pseudoaneurysms—are treated with surgical intervention. Although open chest revascularization is a widely performed procedure, it still carries high risk of morbidity and mortality, especially in second thoracotomy patients, such as the anastomosis pseudoaneurysms secondary to the Bentall procedure of our case [5,9]. In the present case, the patient underwent a Bentall procedure previously, which made open repair more difficult surgically than common cases. Therefore, these patients may benefit from a less invasive approach using endovascular techniques and stent grafts.

TEVAR has become a widely used treatment for descending aortic lesions in recent yaers [4]. However, owing to anatomic limitations and physiologic characteristics, TEVAR has not been widely applied in ascending aortic pathologies [10]. The stent graft should fixate well and seal proximally just distal to the sinotubular junction. Distal landing should be obtained just proximal to the innominate artery. Any inaccurate placement of the stent graft can lead to lethal complications, such as coronary artery or superior branch of aortic arch obstruction. On the other hand, migration forces in the ascending aorta are greater than the stent grafts placed in the descending and abdominal aorta; the devices used for the descending thoracic aorta have size and design limitations that make their application to the ascending aorta difficult [11]. Therefore, the ascending aorta is the final segment of the aorta to be explored with endovascular repair. Recently, owing to less traumatic and faster recovery profile, technological developments have enabled the tentative use of TEVAR for the treatment of the ascending aorta, and it was found to be a treatment option in selected high-risk patients [12,13]. In the present case, a shortened graft and modified delivery system made successful the precise landing of the stent graft in the coronary and brachiocephalic artery opening [14].

RVP originates from balloon valvuloplasty and valve deployment and has been used to reduce blood vessel tension in transcatheter aortic valve valvuloplasty and replacement [7,15,16]. Compared to pharmacological methods, RVP is an ideal technique to temporarily stop the heart from pumping and enable a quick return. RVP produces more profound hypotension more quickly and with shorter duration. It not only reduces heart motion and prevents dislocation during release but also offers an instant reversal of hemodynamics. Due to the greater migration force, transient stopping of cardiac output might be particularly beneficial to these procedures during stent release and anchoring in the ascending aorta [4,5]. However, unrevascularized coronary artery disease, severe valvular heart disease, advanced systolic or diastolic dysfunction, severe pulmonary hypertension or right heart failure, and frequent nonsustained ventricular tachycardia may have the potential for hemodynamic instability after RVP [6]. In the present case, we employed a balloon-tipped floating catheter to induce RVP, producing low cardiac output to facilitate precision landing and stent graft release. After the maneuver, hemodynamics recovered immediately, avoiding prolonged low blood pressure and circulatory collapse.

## 4 Conclusions

TEVAR assisted by RVP is a promising combination for ascending aortic pseudoaneurysm in selected patients. However, its clinical merits warrant further investigation.

## Production notes

### Author contribution statement

All authors listed have significantly contributed to the investigation, development and writing of this article.

### Data availability statement

No data was used for the research described in the article.

### Additional information

No additional information is available for this paper.

## Declaration of competing interest

The authors declare that they have no known competing financial interests or personal relationships that could have appeared to influence the work reported in this paper.

## References

[1] Guo M.H., Appoo J.J., Saczkowski R. (2018). Association of mortality and acute aortic events with ascending aortic aneurysm: a systematic review and meta-analysis. JAMA Netw. Open.

[2] Muetterties C.E., Menon R., Wheatley G.H. (2018). A systematic review of primary endovascular repair of the ascending aorta. J. Vasc. Surg..

[3] Ghoreishi M., Shah A., Jeudy J. (2020). Endovascular repair of ascending aortic disease in high-risk patients yields favorable outcome. Ann. Thorac. Surg..

[4] Saadi E.K., Tagliari A.P., Almeida R.M.S. (2019). Endovascular treatment of the ascending aorta: is this the last frontier in aortic surgery?. Braz. J. Cardiovasc. Surg..

[5] Klonaris C., Georgopoulos S., Katsargyris A. (2016). Endovascular treatment of the ascending aorta: new frontiers for thoracic endovascular aneurysm repair?. J. Thorac. Dis..

[6] Bokoch M.P., Hiramoto J.S., Lobo E.P. (2017). Rapid ventricular pacing for landing zone precision during thoracic endovascular aortic arch repair: a case series. J. Cardiothorac. Vasc. Anesth..

[7] Fefer P., Bogdan A., Grossman Y. (2018). Impact of Rapid ventricular pacing on outcome after transcatheter aortic valve replacement. J. Am. Heart Assoc..

[8] Jones B.M., Jobanputra Y.A., Krishnaswamy A.A. (2020). Rapid ventricular pacing during transcatheter valve procedures using an internal device and programmer: a demonstration of feasibility. Cathet. Cardiovasc. Interv..

[9] Ahmadi H., Shirani S., Jam M.S. (2011). Huge ascending aortic pseudoaneurysm 13 Years after bental surgery with tube graft. Cardiol. J..

[10] Skripochnik E., Ford B., Bilfinger T.V. (2020). Endovascular repair of the ascending aorta for an anastomotic saphenous vein graft aneurysm. Ann. Vasc. Surg..

[11] Preventza O., Le Huu A.L., Olive J. (2022). Endovascular repair of the ascending aorta: the last frontier. Ann. Cardiothorac. Surg..

[12] Tsilimparis N., Drewitz S., Detter C. (2019). Endovascular repair of ascending aortic pathologies with tubular endografts: a single-center experience. J. Endovasc. Ther..

[13] Hsieh Y.K., Lee C.H. (2019). Experience of stent-graft repair in acute ascending aortic syndromes. J. Card. Surg..

[14] Valvo R., Costa G., Barbanti M. (2019). How to avoid coronary occlusion during TAVR valve-in-valve procedures. Front Cardiovasc Med.

[15] Kleczynski P., Dziewierz A., Socha S. (2020). Direct Rapid left ventricular wire pacing during balloon aortic valvuloplasty. J. Clin. Med..

[16] Stąpór M., Trębacz J., Ł Wiewiórka (2020). Direct left ventricular wire pacing during transcatheter aortic valve implantation. Kardiol. Pol..

